# Preparation of calcium carbonate microrods from the gypsum scale layer of evaporation equipment

**DOI:** 10.1039/d2ra00372d

**Published:** 2022-04-06

**Authors:** Xinsong Yuan, Xiaolong Chen, Shan Gao, Yanping Wang, Liang Yang, Qi Zhang, Yiwen Chen, Bainian Wang, Baojun Yang

**Affiliations:** School of Chemistry and Chemical Engineering, Hefei Normal University Hefei 230601 China; School of Chemistry and Chemical Engineering, Hefei University of Technology Hefei 230009 China yuanxs@zju.edu.cn bj_yang@hfut.edu.cn

## Abstract

The difficult-to-remove CaSO_4_ scale layer attached to an evaporator wall is a major problem in related industries. How to efficiently remove the CaSO_4_ scale layer and convert it into fine chemicals with high added value, so as to turn waste into treasure, is a current research hotspot. In this study, a CaSO_4_ scale layer was removed by 15 min rotary washing *via* a phase transfer route. Further, using the eluted calcium gluconate solution as a raw material and polyethylene glycol as the crystal control agent, CaCO_3_ was prepared by a CO_2_ carbonization method. The preparation conditions of CaCO_3_ were optimized by single factor experiments, and the phase and morphology of the prepared samples were characterized by XRD and FESEM. The results show that the optimized conditions are as follows: reaction temperature 80 °C, reaction time 1 h, polyethylene glycol addition 3%, and a stirring rate of 400 rpm. The samples prepared under these conditions are pure-phase calcite-type CaCO_3_ microrods with lengths of 1–2 μm and diameters of 300–500 nm.

## Abstract 2

The difficult-to-remove CaSO_4_ scale layer attached to an evaporator wall is a major problem in related industries.

## Graphical abstract

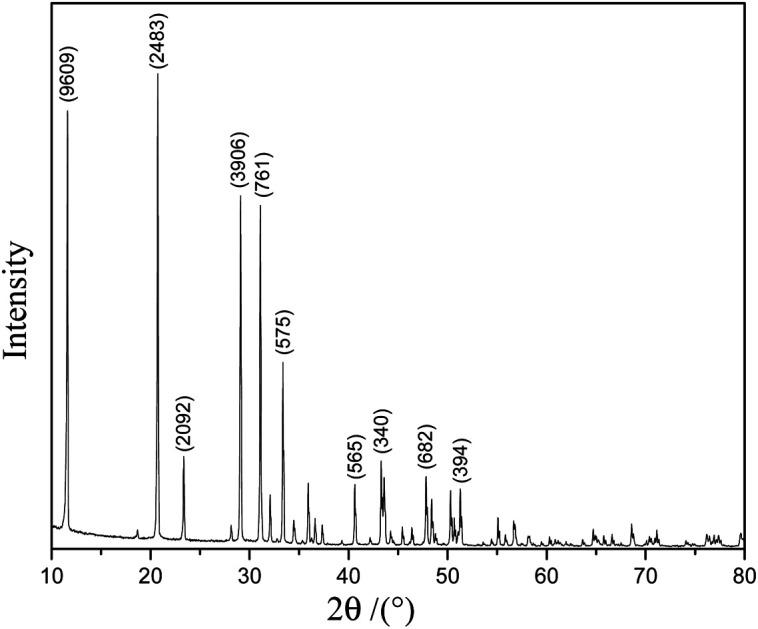

## 1 Introduction

The use of industrial and agricultural wastes to produce high value-added chemical products is an inherent requirement for realizing atomic economy and green chemistry. Scaling is a common problem faced by water treatment processes such as seawater desalination and evaporation and concentration of high-salt wastewater. In chemical engineering processes including the evaporative concentration of lime-treated hemicellulose dilute sulfuric acid hydrolysate,^1,2^ and the distillation of ammonia^3^ in the ammonia-alkali system, a large amount of gypsum waste residue is generated, and the hard-to-remove CaSO_4_ scale layer on the evaporator wall is a major problem. These CaSO_4_ form an insulating layer, reduce evaporation efficiency, waste steam, and reduce equipment utilization. This kind of gypsum scale layer is difficult to remove by common chemical methods, and can only be removed by mechanical methods. Mechanical treatment is not only labor-intensive, but also easy to cause damage to equipment, and at the same time produces a large amount of gypsum waste residue, which is a major disadvantage of lime treatment. In addition, the accumulation of these CaSO_4_ forms a large amount of solid waste residue, which is likely to cause environmental pollution problems.

CaCO_3_ powder is an excellent inorganic filler and reinforcing agent with stable chemical properties and low cost. Due to its diverse polymorphic forms, CaCO_3_ has broad application prospects in the fields of plastics, rubber, coatings, adhesives, papermaking and cosmetics.^4–8^ It is known that the performances of materials are closely related to their size, morphology and composition.^9–12^ The preparation of crystals with specific sizes and morphologies by controlling synthesis conditions is a hotspot in the field of functional material research. Vaterite-type CaCO_3_ has a particularly large specific surface area and high porosity,^13,14^ which has a great potential as a drug carrier with high loading.^15,16^ As a new type of filler, rod-shaped precipitated CaCO_3_ has good strengthening and toughening effects on rubber and plastics. In the paper industry, compared with heavy and nano-calcium carbonate, CaCO_3_ microrods as paper filler has better retention rate, smaller reduction rate of paper strength, and higher covering rate.^4^ As a paper filler and coating, CaCO_3_ microrods also impart excellent printability to paper. As a paper filler and coating, rod calcium carbonate also imparts excellent printability to paper.^17^ Therefore, the technical research and production of rod-shaped calcium carbonate has good economic and social benefits. Herein, we report a method for removing the gypsum scale layer of an evaporator by a phase transfer method,^18^ and a process for preparing calcite-type CaCO_3_ microrods by CO_2_ carbonization using this phase transfer liquid as a raw material.

## 2 Experimental

### 2.1 Sample preparation

#### 2.1.1 Removal of CaSO_4_ scale layer in rotary flask

In the pre-treatment process before fermentation, the dilute sulfuric acid hydrolysate of hemicellulose is neutralized with lime. The hydrolysate was then evaporated and concentrated using a 10 L rotary evaporation flask, which resulted in the formation of a CaSO_4_ scale layer on the inner wall.^19^ The pH value of the concentrated hydrolyzate is about 6.5. 39.3 g sodium gluconate was dissolved in 300 mL of deionized water and added to the evaporator. The flask was spun and washed at 60 rpm for 15 min. It was beneficial to increase the phase transfer rate by lightly brushing the walls with a brush. The phase transfer solution (calcium gluconate) was obtained by filtration.

#### 2.1.2 Synthesis of CaCO_3_

The concentration of calcium gluconate was analyzed by chemical titration. 100 mL of 0.6 mol L^−1^ calcium gluconate solution was prepared from the above phase transfer solution, and was then transferred to a 250 mL round-bottom flask. 4.8 g of NaOH was added to the phase transfer solution to adjust the solution to an alkaline environment to improve the CaCO_3_ yield. An appropriate amount of polyacrylamide (PAM), cetyltrimethylammonium bromide (CTAB), polyethylene glycol (PEG), or aluminum chloride (AC) was used as crystal control agents, and calcium carbonate microrods were prepared by CO_2_ carbonization. The influence of the factors including reaction temperature, reaction time, stirring rate, the type and addition amount of the crystal control agent on the morphology of the synthesized samples was studied by single factor experiments.

### 2.2 Characterization

#### 2.2.1 XRD

The composition and phase purity of the samples were characterized by X-ray diffraction (XRD) on an X-Pert PRO MPD X-ray diffractometer (Cu-Kα target, tube voltage: 40 kV) from PANalytical, the Netherlands.

#### 2.2.2 FESEM

The morphology of the samples was observed with a SU8020 cold field emission scanning electron microscope (scanning voltage set to 15 kV) manufactured by Hitachi, Japan. Before the test, a small amount of the prepared sample was dispersed in an ethanol solution under the action of ultrasonic waves, and a small amount of the ultrasonically dispersed suspension was added dropwise to a clean silicon wafer. After drying, it was adhered to the conductive adhesive to enhance the conductivity, and the morphology of the samples at different times was observed.

## 3 Results and discussion

### 3.1 Phase and morphology analysis of evaporation flask scale layer

**Fig. 1:**
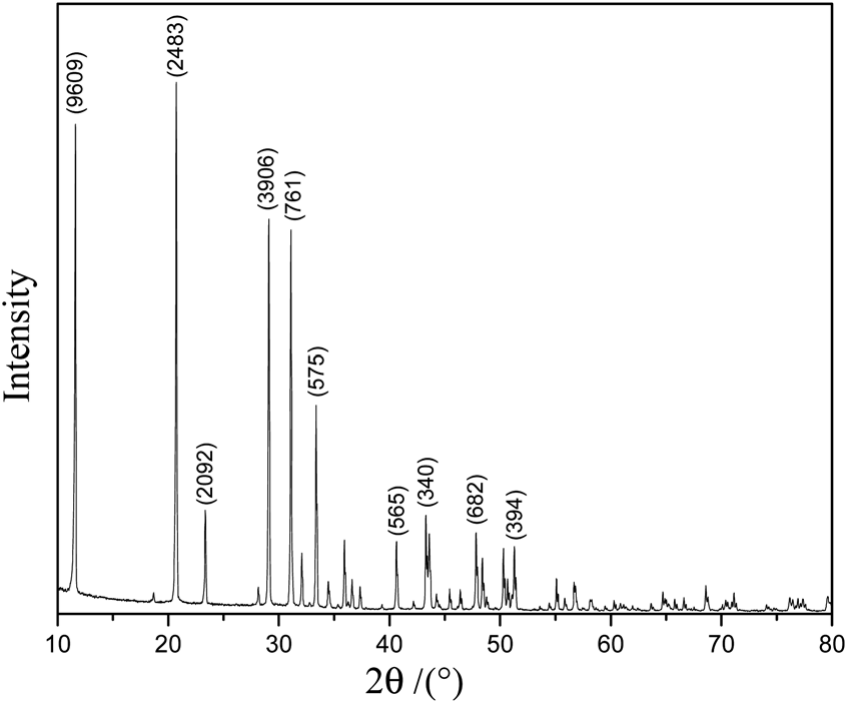
XRD pattern of the scale layer on the inner wall of the evaporating flask.

Fig. 1 shows the XRD pattern of the scale layer on the inner wall of the evaporating flask during the concentration of the lime-neutralized hydrolysate. All the strong and sharp diffraction peaks of the XRD pattern can be indexed as the monoclinic phase CaSO_4_·2H_2_O (JCPDS No. 33-0311), which shows that the main component of the precipitate from the lime neutralized hydrolysate is gypsum.

**Fig. 2:**
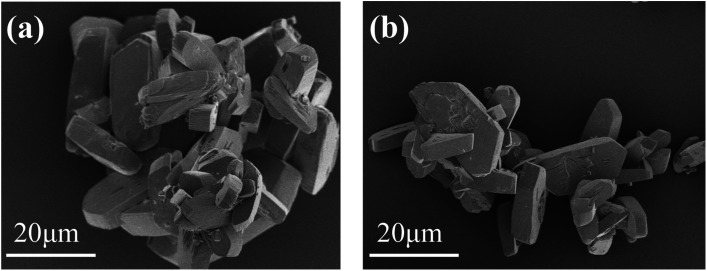
FESEM image of the scale layer sample of the evaporation flask: typical morphology of agglomerated bulk particles (a); morphology of relatively dispersed region (b).

In order to further analyze the morphology of the evaporation flask scale layer samples, we performed FESEM analysis on the samples. It can be seen from Fig. 2a and b that most of the samples are agglomerated cuboid block particles with particle sizes ranging from several microns to tens of microns.

### 3.2 The effect of phase transfer agent on removing scale layer

**Fig. 3:**
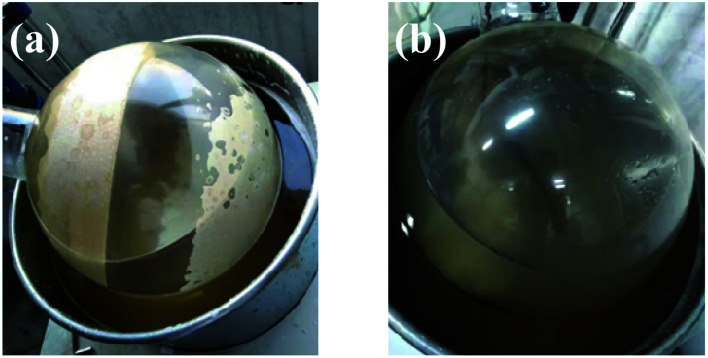
The photo of scaling on the inner wall of the rotary flask (a) and after washing with sodium gluconate solution for 15 min (b).

Using sodium gluconate as the phase transfer agent, the gypsum scale layer on the inner wall of the evaporating flask was completely removed after rotary washing for 15 minutes (as shown in Fig. 3).

In order to completely remove the scale layer, the sodium gluconate was in appropriate excess to the stoichiometric ratio, to a molar ratio of sodium gluconate per Ca^2+^ of 3.0. In this study, the amount of Ca^2+^ was determined based on the amount of calcium gluconate determined in subsequent chemical titration. However, because it is difficult to accurately predict the amount of CaSO_4_ in industry, the amount of calcium gluconate needs to be roughly estimated according to the thickness and area of the scale layer.

We have compared and analyzed the phase transfer method and sonication, base wash and soaking in carbonate or organic solution followed by acid wash. It is inconvenient to sonicate the CaSO_4_ scale layer adhering to the walls of industrial evaporators. The solubility of CaSO_4_ in aqueous solution is very small, and its chemical properties are relatively stable, and the effect of acid washing/alkali washing is not good. In addition, high concentrations of acid and alkali are easy to corrode equipment. Acids tend to corrode metals, and alkalis tend to corrode glass. Sodium citrate, sodium gluconate and EDTA are all commonly used chelating agents, and we compared their treatment effects through experiments. Under the same experimental conditions for 15 minutes, sodium gluconate can completely remove the CaSO_4_ scale layer, sodium citrate can remove about 90% of the CaSO_4_ scale layer, and EDTA can only remove about half. Therefore, sodium gluconate was chosen as the phase transfer agent in this study.

### 3.3 Influence of synthesis conditions on the crystal form and morphology of the prepared CaCO_3_ samples

#### 3.3.1 Influence of crystal control agent type

**Fig. 4:**
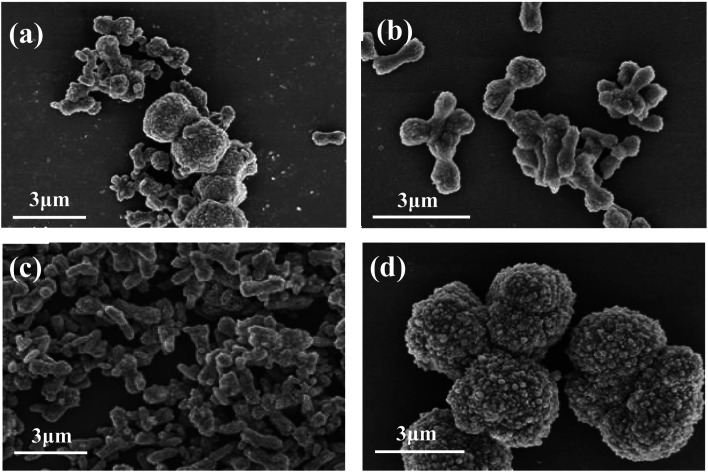
FESEM images of CaCO_3_ samples prepared with PAM (a), CTAB (b), PEG (c) and AC (d) as crystal control agents, respectively.

Other conditions were controlled as follows: reaction temperature 80 °C, reaction time 1.0 h, stirring speed 200 rpm and the addition of the crystal control agents 3% (mass ratio to theoretical yield of CaCO_3_). Fig. 4 is the FESEM images of CaCO_3_ samples generated with different kind of crystal control agents. When PAM (Fig. 4a) was added as a crystal control agent, the obtained samples were mainly short rods with uneven size. When CTAB (Fig. 4b) was used, the obtained samples were mainly dumbbell-shaped short rods. When PEG (Fig. 4c) was added, the obtained samples were basically short rods with a diameter of 500–800 nm and a length of 1–2 μm, and the morphology and dispersion were relatively uniform. When AC (Fig. 4d) was added, the morphology of the obtained sample was mainly microspheres formed by particles. Therefore, PEG was chosen as the crystal control agent in the follow-up experiments.

#### 3.3.2 Influence of reaction temperature

3% PEG was added as the crystal control agent, the stirring speed was 200 rpm and the reaction time was 1.0 h. The effects of the reaction temperature (20 °C, 40 °C, 60 °C and 80 °C) on the crystal form and morphology of the prepared samples were investigated.

**Fig. 5:**
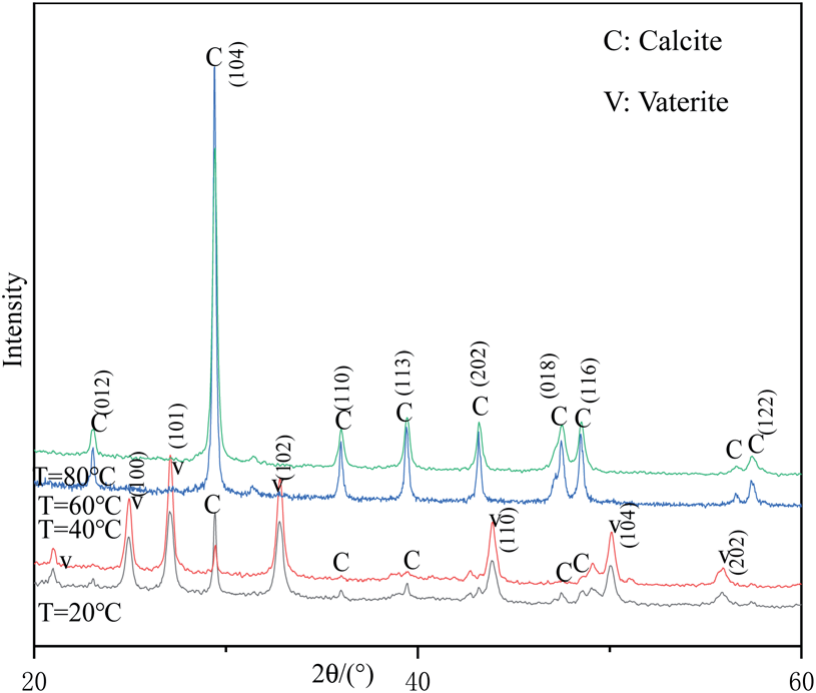
XRD patterns of samples prepared at different reaction temperatures.

Fig. 5 shows the XRD patterns of samples prepared at different reaction temperatures. When the reaction temperature is 60 °C or 80 °C, all the strong peaks in the figure can be indexed as the diffraction peaks of calcite-type CaCO_3_ (JCPDS 99-0022), indicating that the samples prepared under these conditions are pure-phase calcite-type CaCO_3_. When the reaction temperature is lower than 40 °C, the diffraction peaks appearing at 24.95°, 27.19°, 32.71°, 43.90°, 50.13°, 55.86° can be indexed as (100), (101), (102), (110), (104) and (202) crystal plane characteristic diffraction peaks of vaterite-type CaCO_3_ (JCPDS 72-0506), respectively. In addition, a strong calcite-type CaCO_3_ (104) crystal plane diffraction peak appeared at 29.30°, indicating that the samples prepared at this temperature were a mixture of vaterite-type and calcite-type CaCO_3_. Therefore, the reaction temperature above 60 °C is beneficial to obtain pure calcite-type CaCO_3_.

**Fig. 6:**
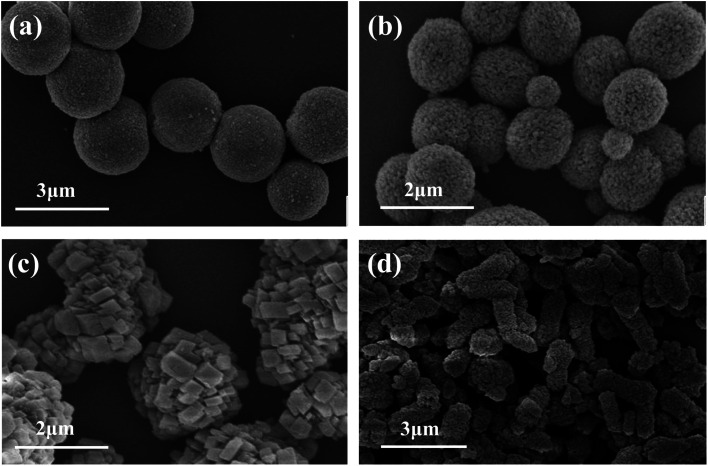
FESEM images of CaCO_3_ samples prepared at different reaction temperatures: (a) 20 °C; (b) 40 °C; (c) 60 °C; (d) 80 °C.

Fig. 6 are the FESEM images of samples prepared at different reaction temperatures. It can be seen that when the reaction temperature is 20 °C (Fig. 6a), the obtained samples are uniform microspheres with particle size of 2–3 μm. When the reaction temperature increased to 40 °C (Fig. 6b), the size and morphology of the sample particles began to change, from the initial uniform spherical shape to different sizes, and the surface became more rough. When the temperature was raised to 60 °C (Fig. 6c), the samples were irregular aggregates formed by many massive or short rod-like small particles. When the reaction temperature was 80 °C (Fig. 6d), the morphology of samples was mainly short rod-like, 1–2 μm long and 500–800 nm in diameter. Therefore, in this experiment, the optimal reaction temperature for preparing pure calcite phase CaCO_3_ microrods is 80 °C.

#### 3.3.3 Influence of reaction time

**Fig. 7:**
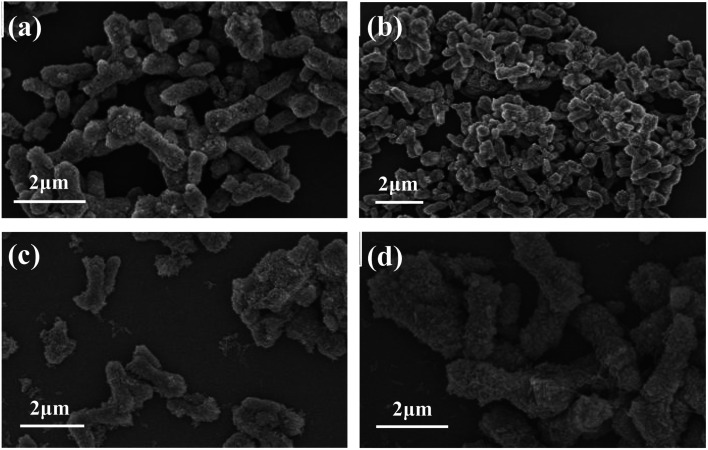
FESEM images of CaCO_3_ samples prepared at different reaction times: (a) 0.5 h; (b) 1.0 h; (c) 1.5 h; (d) 2.0 h.

3% PEG was used as the crystal control agent, the stirring rate was controlled at 200 rpm and the reaction temperature was 80 °C, and the effect of reaction time (0.5 h, 1.0 h, 1.5 h and 2.0 h) on the morphology of the samples was investigated. The experimental results are shown in the Fig. 7. It can be seen from the figure that when the reaction time is 0.5 h (Fig. 7a), most of the obtained samples are rod-shaped, but the sample sizes are not uniform. When the reaction time was extended to 1.0 h (Fig. 7b), the obtained samples were mainly rod-shaped, with an increased aspect ratio and a clearer surface profile of the rods. When the aging reaction time was increased to 1.5 h and 2 h (Fig. 7c and d), the sample still showed a rod-like morphology, but the surface contour of the rod was unclear and accompanied by the formation of small particles. Therefore, the suitable reaction time is 1.0 h.

#### 3.3.4 Influence of PEG addition amount

PEG was selected as the crystal control agent, the stirring rate was 200 rpm, the reaction temperature was 80 °C and the reaction time was 1.0 h. The influence of the PEG addition amount (1%, 2%, 3%, 4% and 5%) on the morphology of the as prepared CaCO_3_ samples was investigated.

**Fig. 8:**
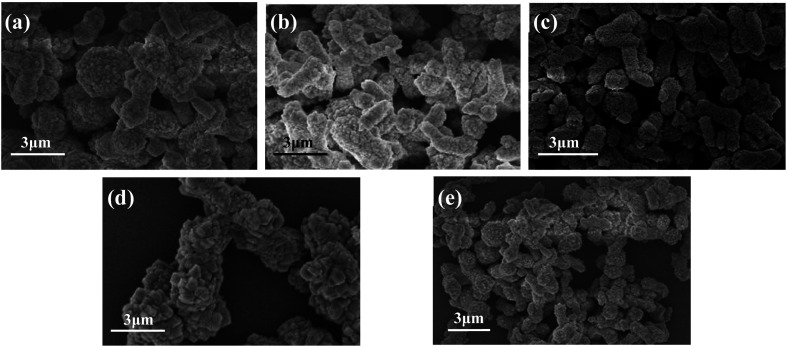
FESEM images of CaCO_3_ samples with different PEG addition amount: (a) 1%; (b) 2%; (c) 3%; (d) 4%; (e) 5%.

Fig. 8 shows the FESEM images of CaCO_3_ samples generated with different addition amount of PEG. When the addition amount was 1% (Fig. 8a), the obtained samples were mainly irregularly agglomerated CaCO_3_ particles, but there were some rod-like structures. When the addition amount was 2% (Fig. 8b), the obtained samples became less agglomerated, the particle size became smaller, and the rod-shaped particles increased. When the addition amount increased to 3% (Fig. 8c), the as prepared CaCO_3_ samples were mainly rod-shaped, the rods are relatively uniform in length and diameter, and the dispersibility was relatively good. When the addition amount increased to 4% and 5% (Fig. 8d and e), the agglomeration of the sample particles was obvious, although some of the particles still maintained rod-like morphology. The experimental results show that the addition of PEG has a significant effect on the morphology of CaCO_3_, and the suitable addition of PEG is 3%.

#### 3.3.5 Influence of stirring speed

**Fig. 9:**
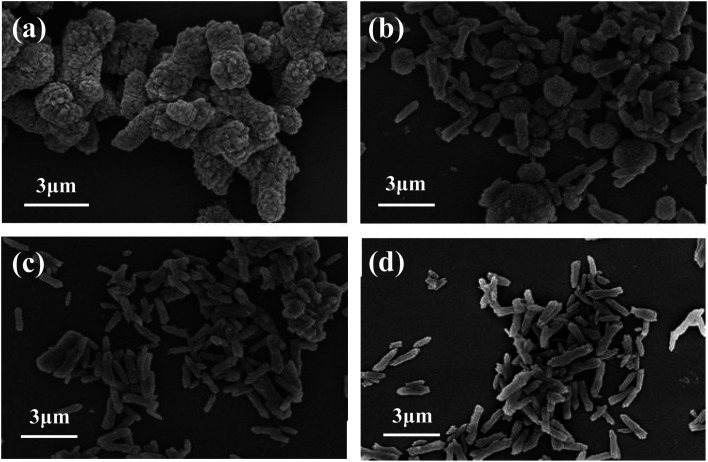
FESEM images of calcium carbonate samples prepared at different stirring rates (a) 200 rpm; (b) 300 rpm; (c) 400 rpm; (d) 500 rpm.

PEG was used as the crystal control agent, the reaction temperature was controlled at 80 °C and the reaction time was 1 h, and the effect of stirring speed (200 rpm, 300 rpm, 400 rpm and 500 rpm) on the morphology of the prepared calcium carbonate was investigated. The experimental results are shown in Fig. 9.

It can be seen from the figure that when the stirring rate is 200 rpm (Fig. 9a), the obtained sample has a rod-like morphology, with a length of 2–3 μm and a diameter of 800–1200 nm, and the particles have obvious agglomeration. When the stirring rate was increased to 300 rpm (Fig. 9b), the obtained sample had a mixed morphology of rod and spherical, but the average particle size of the sample became smaller and the aspect ratio increased. When the stirring rate reached 400 rpm (Fig. 9c) and 500 rpm (Fig. 9d), the obtained samples were mainly short rods with a length of 1–2 μm and a diameter of 300–500 nm, the particle surface was relatively smooth, and the dispersion was good. The above experimental results show that the stirring rate has an effect on the size and morphology of the obtained CaCO_3_ samples. When the stirring rate is relatively low, the generated sample particles are large and the agglomeration is obvious. When the stirring rate increases, the obtained sample particles become smaller, the surface becomes smoother, the size and morphology of the short rod-shaped particles are uniform, and the dispersibility is also better. Therefore, the appropriate stirring rate was determined to be 400 rpm.

The weight of the calcium sulfate scale was calculated based on the change in the weight of the evaporating flask before and after the phase transfer treatment. Under the above optimized conditions, 11.8 g CaCO_3_ was obtained from 26.5 g dry scale. Therefore, the yield of the process based on Ca^2+^ was about 76.7%.

**Fig. 10:**
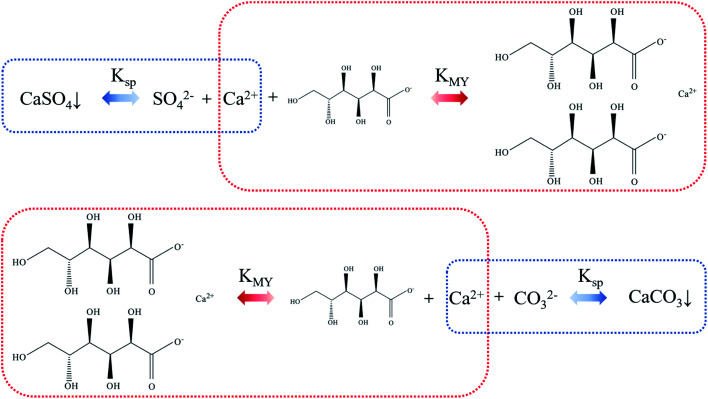
Schematic diagram of complexation and dissociation between gluconate and Ca^2+^.

As regard to the formation of calcium carbonate in this study, the possible mechanism was speculated as follows. Sodium gluconate is easily soluble in water and has strong chelating ability for Ca^2+^, Mg^2+^ and Fe^2+^, so it can efficiently remove the gypsum scale layer on the evaporator wall. The solubility product constant K_sp_ of CaSO_4_ is less than the stability constant K_MY_ of calcium gluconate. Through the control of reaction conditions, the equilibrium of the mixed solution of SO_4_^2−^, Ca^2+^ and gluconate moves to the direction of forming calcium gluconate complex. After the stable complex is formed, its ion and hydration radius increase sharply, and the solubility is better, thereby forming a soluble calcium gluconate solution, which makes the scaled calcium sulfate in the flask continuously dissolve. Under alkaline conditions, CO_2_ was introduced into the calcium gluconate solution, so that the CO_3_^2−^ concentration in the system was continuously increased. Since K_MY_ of calcium gluconate complex is smaller than K_sp_ of CaCO_3_, the balance of the mixed system of CO_3_^2−^, Ca^2+^ and calcium gluconate is constantly moving towards the direction of CaCO_3_ precipitation. The schematic diagram is shown in Fig. 10.

PEG is a water-soluble, non-toxic surfactant, which has a great influence on the morphology of crystals such as CaCO_3_ and Ca_3_(PO_4_)_2_. PEG is a polymer containing hydrophilic groups and its structure is a zigzag long chain. The “ether bond” oxygen atom (–O–) on the chain has unbonded lone pair electrons, which have high electronegativity and are easy to combine with Ca^2+^ ions, so that PEG has abundant CaCO_3_ along the main chain direction crystal nucleation sites. In addition, during the growth of CaCO_3_ crystals, the steric hindrance of chelated calcium ions is large,^20^ which makes CaCO_3_ susceptible to the specification of PEG chains and grows along a specific direction, and thus is beneficial to the formation of CaCO_3_ rods.

## 4 Conclusions

CaSO_4_ scale layer was removed by 15 min rotary washing by using sodium gluconate as the phase transfer agent, which provides an efficient technical route for industrial removal of gypsum scale layer on the evaporator wall. Using the eluted calcium gluconate as raw material and polyethylene glycol as crystal control agent, the process conditions for preparing CaCO_3_ by CO_2_ carbonization were studied by single factor experiments. The samples prepared under the optimal conditions are pure-phase calcite-type CaCO_3_ microrods with length of 1–2 μm and diameter of 300–500 nm.

## Conflicts of interest

There are no conflicts to declare.

## Supplementary Material
